# Comparison of Treatment Outcomes of New Smear-Positive Pulmonary Tuberculosis Patients by HIV and Antiretroviral Status in a TB/HIV Clinic, Malawi

**DOI:** 10.1371/journal.pone.0056248

**Published:** 2013-02-15

**Authors:** Hannock Tweya, Caryl Feldacker, Sam Phiri, Anne Ben-Smith, Lukas Fenner, Andreas Jahn, Mike Kalulu, Ralf Weigel, Chancy Kamba, Rabecca Banda, Matthias Egger, Olivia Keiser

**Affiliations:** 1 The International Union Against Tuberculosis and Lung Disease, Paris, France; 2 Lighthouse Trust, Lilongwe, Malawi; 3 International Training and Education Center for Health, University of Washington, Seattle, Washington, United States of America; 4 Department of Biomedical Informatics, University of Pittsburgh, Pittsburgh, Pennsylvania, United States of America; 5 Institute of Social and Preventive Medicine, University of Bern, Bern, Switzerland; 6 Central Monitoring and Evaluation Division/Department for HIV and AIDS, Ministry of Health, Lilongwe, Malawi; 7 Disease Control Strategy group, Liverpool School of Tropical Medicine, Liverpool, United Kingdom; 8 Ministry of Health, Lilongwe District Health Office, Lilongwe, Malawi; Temple University School of Medicine, United States of America

## Abstract

**Background:**

Smear-positive pulmonary TB is the most infectious form of TB. Previous studies on the effect of HIV and antiretroviral therapy on TB treatment outcomes among these highly infectious patients demonstrated conflicting results, reducing understanding of important issues.

**Methods:**

All adult smear-positive pulmonary TB patients diagnosed between 2008 and 2010 in Malawi’s largest public, integrated TB/HIV clinic were included in the study to assess treatment outcomes by HIV and antiretroviral therapy status using logistic regression.

**Results:**

Of 2,361 new smear-positive pulmonary TB patients, 86% had successful treatment outcome (were cured or completed treatment), 5% died, 6% were lost to follow-up, 1% failed treatment, and 2% transferred-out. Overall HIV prevalence was 56%. After adjusting for gender, age and TB registration year, treatment success was higher among HIV-negative than HIV-positive patients (adjusted odds ratio 1.49; 95% CI: 1.14–1.94). Of 1,275 HIV-infected pulmonary TB patients, 492 (38%) received antiretroviral therapy during the study. Pulmonary TB patients on antiretroviral therapy were more likely to have successful treatment outcomes than those not on ART (adjusted odds ratio : 1.83; 95% CI: 1.29–2.60).

**Conclusion:**

HIV co-infection was associated with poor TB treatment outcomes. Despite high HIV prevalence and the integrated TB/HIV setting, only a minority of patients started antiretroviral therapy. Intensified patient education and provider training on the benefits of antiretroviral therapy could increase antiretroviral therapy uptake and improve TB treatment success among these most infectious patients.

## Introduction

Approximately one third of the world’s population is infected with tuberculosis (TB) bacilli and at risk of developing active TB [1]. In 2010, there were an estimated 12 million TB cases, including 8.8 million incident cases [1]. Smear-positive pulmonary TB (PTB) constitutes 34% of new TB cases [2] and is most likely a source of TB transmission in the community. In sub-Saharan Africa, high HIV prevalence increases the risk of developing TB. An estimated 40% of African TB cases were HIV co-infected in 2010, and 24% of 1.45 million TB deaths globally were among HIV-infected people [1].

Due to the high HIV prevalence among TB patients, WHO recommends the Three *I’s*: intensified TB screening among HIV-infected individuals, provision of isoniazid preventive therapy (IPT), and infection control [3]. Adoption of the comprehensive service package, in particular provision of IPT, by the Malawi national HIV programme was a challenge due to its implementation requirements. However, Malawi’s national HIV programme started providing IPT among pre-ART patients in 2011 [4].

Treatment success in TB patients is a major challenge in TB programmes. TB treatment outcomes (cured, treatment completed, died, treatment failure, lost to follow-up (LTFU) or transferred-out) are known to be influenced by a number of factors including HIV co-infection, but the evidence on what factors are most influential appears inconclusive. While some studies found lower TB cure rates among TB/HIV patients [5], [6], other studies reported comparable TB cure rates among those TB/HIV co-infected to those infected only with TB [7], [8]. Most of these studies were limited by a number of factors. First, they were conducted largely prior to the widespread use of ART [5]–[6], which improves survival [9]. Second, other studies showed reduction in mortality among HIV-infected TB patients who accessed antiretroviral therapy (ART) [10]–[12]; however, these studies focused on ART and not TB outcomes, reducing their application to strengthen TB programmes.

Understanding TB treatment outcomes of new smear-positive PTB cases may provide an indication of the effectiveness of national TB programmes. We, therefore conducted a study to explore TB treatment outcomes of new smear-positive adult PTB cases among those who accessed treatment at Malawi’s largest public TB registration site, Martin Preuss Centre (MPC), in the capital, Lilongwe. MPC integrates TB and ART services. Therefore, we were able to stratify by HIV and ART status to understand better the role of these critical factors.

## Methods

### Setting

The study was conducted at the Martin Preuss Centre (MPC), an integrated TB/HIV clinic run by the Malawi Ministry of Health's Lilongwe District Health Office in partnership with the Lighthouse Trust. MPC, described in detail previously [13], has three units: HIV testing and counseling, ART, and TB which includes sputum submission. “Opt-out” HIV testing and counseling services are also provided within the TB unit; over 95% know their HIV status before starting TB treatment. MPC registers approximately 3,200 TB patients annually. Diagnosis of TB is based on clinical examination, sputum smear microscopy, chest radiography and other investigations as appropriate for extra-pulmonary disease. Once diagnosed with TB, patients are recorded in the national TB register by the district TB officer. The Malawi National TB control (NTP) program classifies new smear-positive PTB patients as, “patients with positive smear result who have never taken anti-tuberculosis drugs for more than one month” [14]. All new adult TB cases are treated with the standard WHO regimen I, consisting of two months of daily rifampicin (R), Isoniazid (H), pyrazinamide (Z) and ethambutol (E), followed by four months of RHE.

Approximately 50% of patients initiate and continue TB treatment at MPC; the rest initiate at MPC but choose to continue their treatment at one of 18 peripheral health facilities in accordance with NTP decentralization efforts. Treatment outcomes of all patients who initiate at MPC regardless of where they continue treatment are recorded and updated using TB treatment cards in the MPC central TB register. The Malawi NTP defines TB treatment outcomes as: 1) cured: a patient who was smear-positive at diagnosis and has smear-negative results at, or one month prior to, completion of TB treatment; 2) treatment completed: a patient who completed treatment (i.e. took a full course of treatment) but for whom smear results are not available on, or one month prior, to the completion of TB treatment; 3) failure: a patient who remains, or returns, smear-positive at five months or more during TB treatment; 4) death: a patient who died from any cause during TB treatment; 5) lost to follow-up: a patient who stopped TB treatment for more than two months; 6) transferred-out: a patient who (presumably) completed TB treatment at another TB registration site. Deaths are ascertained mainly through active follow-up.

MPC also provides ART to HIV-infected individuals. All TB patients diagnosed with HIV were eligible for ART and expected to initiate ART within two months of TB registration. ART was co-administered with TB treatment and available for all patients regardless of where they chose to continue TB treatment.

### Study Design and Population

We included all new smear-positive adult PTB patients (≥15 years) who registered and initiated TB treatment at MPC between January 2008 and December 2010. Patients who completed TB treatment at MPC or a peripheral site were included.

### Data Collection and Data Analysis

Variables for all new smear-positive PTB cases were collected from routine programme data including TB registers and patient treatment cards. Variables included TB registration number, registration date, age, gender, HIV status, ART status and TB treatment outcome. Data were entered and cleaned in a MS Access database and analyzed in STATA 10.0. We used chi-square tests to compare patient characteristics and all TB treatment outcomes stratified by HIV and ART status. TB cases with missing treatment outcomes were excluded from further analysis. The effects of HIV and ART status on TB treatment outcome were examined using logistic regression models. TB treatment outcome was categorised as treatment success (“cured” or “treatment completed”) versus all other treatment outcomes. The final multivariable model was determined using forward selection, including explanatory variables with a two-sided p-value of ≤0.05. Age group, in 10-year bands, and gender were included a priori in the final multivariable logistic regression model. Linear trends for treatment success were explored using chi-square tests. Statistical significance for all analyses was set at the p≤0.05 level and 95% confidence intervals (CI) were calculated throughout.

### Ethical Considerations

The study was approved by the Malawi National Health Science Research Committee and the Ethics Advisory Group of the International Union Against Tuberculosis and Lung Disease in Paris, France. The data for the study did not include any personal identifiers. The ethics committees waived the need for patient consent because the study used routine programmatic data that did not include any personal identifiers.

## Results

### Study Population

Between January 2008 and December 2010, 2,478 new, smear-positive, adult PTB cases were registered at MPC. Men comprised 62% (1,530) of all patients. The median age was 31 years (Interquartile range (IQR) 26–38 years). There were no age differences across years of TB registration. A total of 117 (5%) patients had unknown TB treatment outcome and were excluded in further analyses. Of the remaining 2,361, 86% had treatment success (1664 were cured and 376 completed treatment), 5% died, 6% were LTFU, 2% transferred-out and 1% had treatment failure **(** **Table 1** **)**. Treatment success decreased from 90% in 2008 to 84% and 86% in 2009 and 2010, respectively (test for trend *p*  = 0.013). The proportions of LTFU were higher in 2009 (10%) and 2010 (6%) compared to 2008 (2%).

**Table 1:** Patient characteristics by TB treatment outcomes for new smear-positive TB patients at Martin Preuss Centre between January 2008 and December 2010.

Characteristics	Total(Column: #, %)		TB treatment outcome															
			Success₣				Died			LTFU*			Transfer out		failure		*P-value*	
**Total**	**2,361**		***2,041***	***86%***		***110***		***5%***	***146***		***6%***	***50***		***2%***	***14***	***1%***		
**Sex**																		*0.010*
Male	1,460	*62%*	1,238	*85%*		*73*		*5%*	*103*		*7%*	39		*3%*	*7*	*1%*		
Female	901	*38%*	803	*89%*		*37*		*4%*	*43*		*5%*	11		*1%*	*7*	*1%*		
**Age**																		*0.030*
15–24	481	*20%*	415	*86%*		*17*		*4%*	*37*		*8%*	*8*		*2%*	*4*	*<1%*		
25–34	1,023	*43%*	893	*87%*		*35*		*3%*	*61*		*6%*	*28*		*3%*	*6*	*1%*		
35–44	525	*22%*	458	*87%*		*27*		*5%*	*30*		*6%*	*8*		*2%*	*2*	*<1%*		
45–54	186	*8%*	155	*83%*		*18*		*10%*	*9*		*5%*	*3*		*2%*	*1*	*1%*		
≥55	146	*6%*	120	*82%*		*13*		*9%*	*9*		*6%*	*3*		*2%*	*1*	*1%*		
**Treatment site** $																		*0.272*
MPC	943	*40%*	801	*85%*		22		*2%*	87		*9%*	26		*3%*	*7*	*1%*		
Other	1,416	*60%*	1,238	*87%*		88		*6%*	59		*4%*	24		*2%*	*7*	*<1%*		
**TB registration year**																		*<0.001*
2008	841	*36%*	753	*90%*		43		*5%*	19		*2%*	16		*2%*	*10*	*2%*		
2009	869	*37%*	729	*84%*		39		*4%*	87		*10%*	12		*1%*	*2*	*<1%*		
2010	651	*28%*	559	*86%*		28		*4%*	40		*6%*	22		*3%*	*2*	*<1%*		
**HIV status** £																		*0.017*
HIV positive	1,275	*56%*	1,086	*85%*		72		*6%*	84		*7%*	28		*2%*	*5*	*<1%*		
HIV negative	989	*44%*	875	*88%*		31		*3%*	56		*6%*	18		*2%*	*9*	*1%*		
**ART status** ¥																		*0.014*
On ART	492	*39%*	437	*89%*		16		*3%*	28		*6%*	8		*2%*	*3*	*1%*		
Not on ART	783	*61%*	649	*83%*	56			*7%*	56		*7%*	20		*3%*	*2*	*<1%*		

^₣^success =  cured or treatment complete,

¥ Included HIV positive only,

£ 97 TB cases had missing HIV status,

$ 2 TB cases had missing treatment site.

* LTFU =  lost to follow-up.

### Comparison of Smear-positive PTB Patient Treatment Outcomes by HIV Status

Most patients (2,264, 96%) knew their HIV status. HIV prevalence was 56%: 60% in women and 54% in men. HIV-negative patients had slightly better TB outcomes compared to HIV-positive patients. Of HIV-negative patients, 88% had successful treatment outcomes compared to 85% of HIV-positive patients. More HIV-infected TB patients died (6%) compared to HIV-negative patients (3%) (p = 0.034) (**Table 2**). In univariable logistic regression analyses, being female (OR  = 1.45, 95% CI 1.12–1.87, p = 0.005) and being HIV-negative (OR 1.34, 95% CI 1.05–1.72, p = 0.019) were both associated with successful TB treatment outcome while age (p = 0.323) and treatment site (p = 0.147) were not associated (**Table 3**). Compared to 2009, treatment success was higher in 2008 (OR = 1.80 95% CI 1.34–2.43) but similar to 2010 (OR = 1.23 95% CI 0.92–1.65). After adjusting for age, gender and TB registration year, treatment success among HIV-negative PTB cases increased compared to HIV-infected peers (adjusted OR = 1.49, 95% CI 1.14–1.94).

**Table 2:** Baseline characteristics and TB treatment outcomes of new smear-positive adult PTB patients by HIV status at Martin Preuss Centre between January 2008 and December 2010.

Characteristics	Total [n (%)]		HIV Positive [n (%)]		HIV Negative [n (%)]		P-value₣
*** Overall***	***2,264***		***1,275***	***56%***	***989***	***44%***	
**Gender**							*0.004*
Male	1,400	*62%*	755	*59%*	645	*65%*	
Female	864	*38%*	520	*41%*	344	*35%*	
**Age at TB registration**							*<0.001*
15–24	460	*20%*	169	*13%*	291	*29%*	
25–34	985	*44%*	619	*49%*	366	*37%*	
35–44	505	*22%*	350	*27%*	155	*16%*	
45–54	173	*8%*	100	*8%*	73	*7%*	
≥55	141	*6%*	37	*3%*	104	*10%*	
**TB Treatment site**							*0.029*
MPC	912	*40%*	533	*42%*	379	*38%*	
Other	1,352	*60%*	742	*58%*	610	*62%*	
**TB registration year**							*0.070*
2008	791	*35%*	421	*33%*	370	*37%*	
2009	843	*37%*	496	*39%*	347	*35%*	
2010	630	*28%*	358	*28%*	272	*28%*	
**TB Treatment outcome** ¥							*0.017*
Treatment success	1,960	*87%*	1085	*85%*	875	*88%*	
Cured	1,601	*82%*	869	80%	732	84%	
Completed	359	*18%*	216	20%	143	16%	
Lost to follow-up	140	*6%*	84	*7%*	56	*6%*	
Treatment failure	14	*<1%*	5	*<1%*	9	*1%*	
Died	103	*5%*	72	*6%*	31	*3%*	
Transfer-out	46	*2%*	28	*2%*	18	*2%*	

¥ 110 TB cases had unknown Tb treatment outcome and treatment success =  cured/completed.

^₣^chi-square test for difference.

**Table 3:** Factors associated with TB treatment success among new smear-positive TB patients at Martin Preuss Centre between January 2008 and December 2010 (N = 2,264)¥.

Characteristics	Total		Unadjusted Odds Ratio(95% CI)	*P-value* *	Adjusted Odds Ratio(95% CI)₣	*P-value* *
	N	%				
**HIV Status**				*0.019*		*0.003*
HIV positive	1,275	*56%*	1.00		1.00	
HIV negative	989	*44%*	1.34 (1.05–1.72)		1.49 (1.14–1.94)	
**Gender**				*0.005*		*0.002*
Male	1,400	*62%*	1.00		1.00	
Female	864	*38%*	1.45 (1.12–1.87)		1.52 (1.17–1.99)	
**Age category**				*0.323*		*0.065*
15–24	460	*20%*	0.90 (0.65–1.25)		0.76 (0.54–1.06)	
25–34	985	*44%*	1.00		1.00	
35–44	505	*22%*	0.98 (0.71–1.36)		1.07 (0.77–1.48)	
45–54	173	*8%*	0.71 (0.46–1.10)		0.70 (0.45–1.10)	
≥55	141	*6%*	0.66(0.41–1.06)		0.57 (0.35–0.93)	
**TB Registration year**				*<0.001*		*<0.001*
2008	791	*35%*	1.80 (1.34–2.43)		1.79 (1.33–2.41)	
2009	843	*37%*	1.00		1.00	
2010	630	*28%*	1.23 (0.92–1.65)		1.22 (0.91–1.63)	
**TB Treatment site**				*0.147*		
MPC	912	*40%*	1.20 (0.94–1.53)		*-*	
Other	1,352	*60%*	1.00			

* P-value for likelihood ratio test,

^₣^Adjusted for sex, age, HIV status and TB registration year,

¥ Treatment success = cured/completed treatment.

### Comparison of Smear-positive PTB Patient Treatment Outcomes by ART Status

Of the 1,275 HIV-infected new smear-positive PTB cases, 492 (38%) received ART during the study period. There were no significant differences in terms of gender and age distributions between those on ART and not. However, the proportion of patients on ART increased from 17% in 2008 to almost 40% in 2010 (p<0.001). Patients on ART had more successful treatment outcomes (89%) compared to those not on ART (83%) (p = 0.014). TB patients who were on ART were less likely to die than those not on ART (adjusted OR  = 0.46 95% CI 0.26–0.83*)*. In multivariable logistic regression, treatment success was higher among smear-positive PTB patients on ART than among those not on ART (adjusted OR  = 1.83, 95% CI: 1.29–2.60) **(** **Table 4** **)**.

**Table 4:** Factors associated with TB treatment success among new smear-positive TB/HIV co-infected patients at Martin Preuss Centre between January 2008 and December 2010 ¥ (N = 1,275).

Characteristics	Total		Unadjusted Odds Ratio(95% CI)	*P-value* *	Adjusted Odds Ratio(95% CI)₣	*P-value* *
	N	%				
**ART Status**				*0.005*		*0.001*
On ART	492	*39%*	1.61 (1.15–2.25)		1.83 (1.29–2.60)	
Not on ART	783	*61%*	1.00		1.00	
**Gender**				*0.031*		*0.032*
Female	520	*41%*	1.43 (1.03–1.97)		1.44 (1.03–2.01)	
Male	755	*59%*	1.00		1.00	
**Age at TB registration**				*0.515*		*0.373*
15–24	169	*13%*	0.76 (0.48–1.19)		0.70 (0.44–1.12)	
25–34	619	*49%*	1.00		1.00	
35–44	350	*27%*	0.98 (0.67–1.43)		1.06 (0.72–1.55)	
45–54	200	*8%*	0.70 (0.40–1.21)		0.73 (0.42–1.28)	
≥55	37	*3%*	1.35 (0.47–3.90)		1.43 (0.49–4.17)	
**TB Registration year**				*0.004*		*<0.001*
2008	421	*33%*	1.90 (1.30–2.79)		2.17 (1.46–3.22)	
2009	496	*39%*	1.00		1.00	
2010	358	*28%*	1.31 (0.91–1.90)		1.22 (0.84–1.78)	

* P-value for likelihood ratio test,

^₣^Adjusted for sex, age, ART status and TB registration year,

¥ Treatment success = cured/completed treatment.

## Discussion

This is one of the largest studies to examine the effects of HIV and ART status on TB treatment outcomes among new smear-positive PTB patients in sub-Saharan Africa. In our study, overall treatment success was 86%, indicating an overall positive programme outcome. Fifty six percent of smear-positive PTB patients were HIV co-infected and HIV co-infection was associated with a slightly poorer TB treatment outcome, even after adjusting for gender, age and year of TB registration. Only 38% of the TB/HIV new smear-positive co-infected patients were on ART. Those on ART had successful TB treatment outcomes compared to those not on ART.

Similar to other studies [15], [16], we found a high proportion of smear-positive PTB among individuals aged 15–44 years; the same ages are at highest risk of HIV in Malawi [17]. However, HIV prevalence among our smear-positive PTB patients (56%) was lower than the national HIV prevalence of 68% among all TB forms [18]. This finding is likely because the national estimation of HIV prevalence in TB-infected individuals includes those with smear-negative PTB, which is quite common among HIV-infected individuals [19].

As expected, both HIV and ART status influenced TB treatment outcomes. In general, HIV-infected individuals were less likely to have successful treatment outcomes and were twice as likely to die as compared to HIV-negative individuals. Among HIV-infected smear-positive PTB adults, those on ART had slightly higher likelihood of successful treatment outcomes compared to those not on ART. ART enhances the immune response among HIV-infected TB patients, and this consequently leads to improved survival [20]. More dramatically, we also found a 50% reduction in mortality among TB patients on ART compared to those not on ART, an indication of the positive effect of TB/ART co-infection treatment, which echoes similar findings from previous studies [9], [21].

Despite the well-known benefit of ART and the integrated TB/HIV care model at MPC, only 38% of HIV-infected new smear-positive individuals received ART during TB treatment. The low ART uptake is likely due to several factors. First, some patients might be reluctant to take ART and TB drugs simultaneously due to concerns about pill burden and drug interactions. Second, the national ART guidelines recommend a guardian before ART initiation; the absence of a guardian may delay or discourage ART uptake among TB patients. Lastly, smear-positive PTB patients are expected to be reviewed by TB clinical officers – a cadre of mid-level healthcare professionals trained in both TB and ART management. However, in practice, health surveillance assistants (the lowest level of healthcare professionals) who are not trained in ART provision often conduct TB reviews for HIV-infected individuals, sometimes overlooking the need for ART initiation [13]. Currently shifting management of smear-positive PTB patients from health surveillance assistants to TB clinical officers is under discussion within the Malawi NTP with the aim of improving routine programme management.

Our results should be viewed in light of the following limitations. TB treatment outcomes were not available for 5% of eligible patients and their exclusion might have introduced bias. However, there were no significant differences in terms of age, gender and HIV status between patients with and without TB treatment outcomes. Second, we used only routine programme data; these do not include information such as viral load and CD4 cell count which may have significant effects on patients’ TB outcomes. Third, since 165 (34%) of HIV-infected individuals on ART did not have a recorded ART start date, we could not explore the effect of duration on ART or timing of ART uptake on TB outcomes. Previous studies found decreased treatment success for sequential ART initiation [9]. Despite these limitations, the study findings are useful to inform policy and programmes that aim to improve management of new smear-positive TB/HIV patients in Malawi and other comparable settings.

The results of this study suggest several changes that would likely improve TB outcomes and reduce the duration of infectiousness especially among TB/HIV co-infected patients in an an integrated TB/HIV clinic setting. First, although our study found satisfactory treatment outcomes for smear-positive PTB patients, this rate decreased from 90% in 2008 to 84% in 2009 and to 86% in 2010, possibly due to changing LTFU rates. In an effort to address LTFU, the Malawi HIV programme recommends active tracing of TB/HIV patients in facilities providing HIV services, a step that MPC will consider. Second, appropriate and intensified sensitization on the benefits of ART for both patients and care providers might increase ART uptake. Lastly, improved monitoring and evaluation of TB/HIV co-infected patient care, especially as provided by health surveillance assistants, would better identify and address obstacles in ART initiation during follow-up visits.
